# Effect of Probiotics Supplementation on Heart Rate: A Systematic Review and Meta-Analysis of Randomized Clinical Trials

**DOI:** 10.3389/fnut.2022.829703

**Published:** 2022-03-22

**Authors:** Shufen Han, Yuezhen Li, Ruijuan Song, Hui Gao, Weiguo Zhang

**Affiliations:** ^1^School of Public Health, Hangzhou Normal University, Hangzhou, China; ^2^Department of Nutrition and Food Hygiene, School of Public Health, Soochow University, Suzhou, China; ^3^Prefecture Center for Disease Control and Prevention, Jiaxing, China; ^4^Independent Researcher, Irving, TX, United States

**Keywords:** probiotics, heart rate, cardiovascular risk factor/disease, randomized controlled trials, meta-analysis

## Abstract

**Background and Aims:**

Probiotics consumption lowers the risk of cardiovascular disease, but whether it affects heart rate (HR) remains controversial. Therefore, our study aimed to assess the chronotropic effects of probiotics on heartbeat via a meta-analysis of randomized clinical trials.

**Methods:**

Relevant studies were identified by searching PubMed, Cochrane library, and Clinical Trials databases up to October 2021. Either a fixed-effects or a random-effects model was used to calculate the pooled effect sizes and 95% confidence intervals (CIs).

**Results:**

This meta-analysis included 13 studies involving 16 interventional trial arms and 931 participants according to inclusion criteria. The overall pooled estimate showed that probiotics supplementation had a slight, but no significant reduction of 0.28 bpm (95% CI: −1.17, 0.60) on HR. Relatively high heterogeneity was observed among included trials (*I*^2^ = 80.8%, *P* heterogeneity < 0.001). Subgroup analysis displayed that probiotics supplementation significantly reduced HR by 2.94 bpm (95% CI: −5.06, −0.82) among participants with baseline HR ≥ 75 bpm, by 1.17 bpm (95% CI: −2.34, −0.00) with probiotics dose ≥1 × 10^10^ CFU/day, and by 1.43 bpm (95% CI: −2.69, −0.17) with multiple-strain intervention. Meta-regression analysis showed that baseline HR was a major potential effect modifier of probiotics supplementation on lowering HR.

**Conclusion:**

Hitherto, the overall evidence in the literature was insufficient to support the notion that probiotics supplementation has a class effect on HR reduction. However, in subgroup analysis, probiotics reduced HR significantly in those who had higher baseline HR, received a higher dose or multiple strains of probiotics.

## Introduction

Heart rate (HR) is determined by cardiac automaticity and highly regulated by the autonomic nervous system that consists of both parasympathetic and sympathetic nerves. Given its role in predicting cardiovascular disease in patients with cardiovascular disease and in the general population, elevated resting HR has been now considered a cardiovascular risk factor (1–3). For example, in a 4-year follow-up of 43,725 participants without metabolic syndrome at baseline, the odds ratio of developing metabolic syndrome was 1.41 over 4 years in those with resting HR of 95–104 bpm compared with 55–64 bpm after adjustment for confounding factors (4). More importantly, mounting evidence has demonstrated that resting HR is a potent predictor of cardiovascular mortality (1, 2). An increase in home-measured resting HR by 5 bpm was associated with a 17% increase in 10-year cardiovascular mortality (5). A recent meta-analysis including 87 prospective studies reported that a greater resting HR with each 10 bpm increment was associated with a 15% higher risk for cardiovascular disease and 18% for heart failure (6). As such, HR has emerged as a potential target for better health outcomes (7, 8).

As suggested by the Food and Agriculture Organization and World Health Organization in 2002, probiotics are live microorganisms that confer a health benefit on the host when consumed in adequate amounts (9). With growing interest in the gut microbiome, probiotics have received considerable attention for human health and wellbeing. A few review articles have shown that the administration of probiotics has certain potentials in producing protective and therapeutic benefits against heart failure and cardiovascular disease by targeting some cardiovascular disease risk factors, such as obesity, hypertension, and dyslipidemia (10–12). The possible mechanisms by which probiotics present their roles are related to the restoration of microbiota diversity, and the reduction of pro-inflammatory molecules and various metabolites. Given the importance of HR in health and disease, it is well-justified to ask whether probiotics supplementation, has a chronotropic effect on the heartbeat in humans. To date, the effect of probiotics supplementation on resting HR has been explored in a fair number of randomized controlled trials (RCTs) (13–25). However, the results appear inconsistent: HR was reported slowing down in some publications, but not in others. Therefore, we performed a meta-analysis and systematic review of RCTs to estimate the effect of probiotics supplementation on HR in different populations and health conditions.

## Methods

### Literature Search Strategy

A systematic literature search for English publications was performed for RCTs to evaluate the effect of probiotics supplementation on HR in the databases of PubMed, Web of Science, and Cochrane library up to October 2021. The following search terms were applied: including “probiotics or probiotic drinks or probiotic pills or probiotic tablets or probiotic capsules or probiotic sachet or probiotic agent or probiotic products or probiotic supplementation or probiotic fortification” combined with “heart rate OR HR OR pulse rate OR PR OR heartbeat”. For additional studies, the bibliographies of retrieved papers and published systematic reviews were also carefully retrieved. We did not attempt to contact the corresponding authors for further information and did not try to take the unpublished articles into consideration. Each search result was independently reviewed for eligibility by two authors, with disagreement resolved by discussion. The present meta-analysis was planned, conducted, and reported in accordance with the preferred reporting items for systematic reviews and meta-analyses guidelines (PRISMA) (26).

### Study Selection

After reviewing the titles and abstracts, full-text manuscripts were screened according to the following inclusion criteria: (1) studies were conducted with human participants with more than 18 years; (2) randomized clinical trial evaluating the effects of probiotics on HR; (3) probiotics was the only active components of the treatment effect; (4) had an intervention duration of not <2 weeks; (5) had a placebo-controlled group; (6) reported the net changes of HR, and their corresponding standard deviations (SDs), or available data to calculate their values. When probiotics were administered for multiple time durations in a study, the results from the longest time were used in the present meta-analysis. The intervention groups taking probiotic-fortified yogurts or probiotic-fortified beverages were included in the present study.

### Data Extraction and Quality Assessment

Two authors (S.H. and Y.L.) extracted the following information from the selected studies via using a standardized data-collection sheet: the first author's name, publication year, country, study design details, sample size, intervention duration, participant characteristics (mean age, sex, and health status), type and dose of probiotics, methods of HR assessment, baseline and final HR, a net change of HR and their corresponding SD. The quality of eligible studies was assessed according to the risk of bias criteria detailed in the Cochrane Handbook for Systematic Reviews of Interventions (The Cochrane Collaboration, 2017). The Jadad scale was used to evaluate the methodological quality of each included trial by assigning scores ranging from 1 to 7 (where <3 indicates low-quality study, 3–4 moderate quality, >4 high-quality) based on a study's randomization, blinding, withdrawals and dropouts (27).

### Data Synthesis and Statistical Analysis

In the present meta-analysis, probiotics were considered as the intervention arm. The net changes in HR were calculated as the difference between final and baseline values in the intervention and control groups, respectively. Studies with no reported SD had their values imputed from standard errors, confidence interval (CI), or *P*-values using a standard formula for the analysis (28). The homogeneity of the effect size among studies was tested using the Cochran Q test at a significance level of *P* < 0.10 level of significance. We also calculated the *I*^2^ statistic, a quantitative measure of inconsistency across studies (29). An *I*^2^ value > 50% was deemed to indicate substantial heterogeneity across trials. In the presence of significant heterogeneity, the random-effects model was used to assess the overall effect size, otherwise, the fixed-effects model was acceptable. A pre-specified subgroup analysis was conducted to determine the possible effects of study designs and participant characteristics on the overall effect size. A sensitivity analysis was performed to investigate the influence of a single study on the overall effect estimate by omitting one study at each turn while pooling the results from the remainder. Furthermore, we performed a meta-regression analysis to explain possible sources of heterogeneity across studies. Potential publication bias was assessed through the Begg funnel plots and the Egger regression test (30). All analyses were performed using STATA version 11.0 (StataCorp, College Station, TX). *P* <0.05 was considered statistically significant, except where otherwise specified.

## Results

### Literature Selection and Trial Characteristics

**Figure 1:**
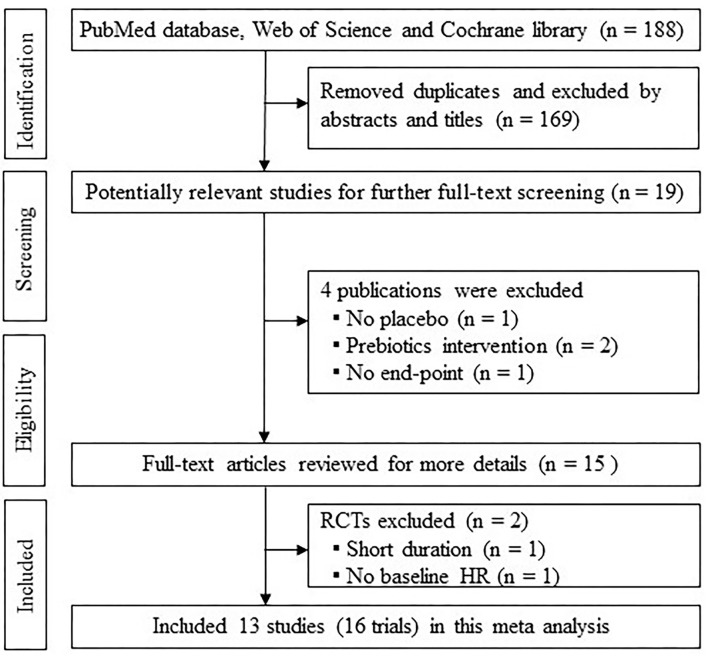
Flow chart of literature selection process.

After the literature search, a total of 188 records were found in our initial literature selection, and 13 eligible studies were incorporated based on study design and inclusion criteria in this meta-analysis (13–25). Among 13 studies, two studies had more than one targeting population (19, 25) and one study had two probiotic species administered separately (23), thereby consisting of 16 intervention arms. A flow chart showing the selection process is presented in Figure 1.

The characteristics of the selected studies including 13 RCTs and their 16 intervention arms are shown in Table 1. The trials were published from 2005 to 2020 by various investigators. The sample size varied from 19 to 120, with a total of 931 participants. In terms of the study design, all the trials had a parallel design and participants received treatment in capsules, tablet, sachet, or a specially fortified yogurt or milk or drink with or without probiotics; one trial was triple-blind, three trials did not report the blindness, and the remaining were double-blind. The duration of probiotics administration ranged from 2 to 16 weeks with a median of 6 weeks. Ten trials used a single strain to intervene, and the remaining used multiple strains. The dose of most probiotics ranged from 3.0 ×10^9^ to 22.5 ×10^10^, and species of probiotics were presented in Table 1. Two studies did not mention the dosage of probiotics (22, 25). Two studies registered pulse rate (15, 25), and the remaining reported HR. One study observed the combined effect of probiotics and 20 mg of iron compared to 20 mg of iron alone (18). For the study from *Patterson*, we selected HR of sitting post-Trier Social Stress Test for 20 min as the final HR (14). For the Jadad scale, 5 individual trials scored 5, 5 trials scored 4, 4 trials scored 3, and 2 trials scored 2.

**Table 1:** Overview and characteristic of the trials and participants in this meta-analysis.

**References (Country)**	**Study design**	Sample size*	**Male (%)**	**Age (year)**	**Duration**	**Probiotic compound**	**Heart rate measurement**	Baseline HR (bpm)*	**Participant characteristics**	**Jadad scores**
Adikari et al. (13) (Malaysia)	P, DB	10/9	100	19.0	8 weeks	*Lactobacillus Casei* Shirota strain with 3 ×10^10^ CFU/d	NeuLog™ Heart Rate sensor	76.0 ± 16.1 77.8 ± 12.2	Football players	5
Patterson et al. (14) (Finland)	P, DB	55/57	50	23.4	5 weeks	*Lacticaseibacillus paracasei* Lpc-37® with 1.75 ×10^10^ CFU/d	A Polar watch device, Polar Electro GmbH	72.3 ± 13.7 71.0 ± 12.4	Healthy adults	5
Karbownik et al. (15) (Poland)	P, DB	31/29	38	22.7	30 days	*Saccharomyces Boulardii* CNCM I-1079 with 5 ×10^9^ CFU/d	Self-recorded by palpation at the radial artery	68.8 ± 9.5 73.8 ± 11.4	Healthy medical students	3
Romão da Silva et al. (16) (Brazil)	P, TB	19/17	0	43.5	8 weeks	^a^Probiatop® at a dosage of 4 ×10^9^ CFU/d	Electrocardiogram	70.0 ± 2.8 74.0 ± 3.6	Hypertensive women	4
Pacifici et al. (17) (Italy)	P, DB	20/20	100	33.5	10 weeks	^b^*Hyperbiotics PRO-15 ADVANCED STRENGHT* with 225 billion CFUs	No report	73.2 ± 7.0 74.2 ± 5.1	Oral surgeons	5
Axling et al. (18) (Sweden)	P, DB	19/23	0	21.9	12 weeks	*Lactobacillus plantarum 299v (Lp299v)* wihh 1 ×10^10^ CFU/d	Polar heart rate monitor with a Polar H7 Sensor	–	Female iron-deficient athletes	3
Ibrahim et al. (19) (Malaysia)	P, NR	10/10	100	22.5	12 weeks	^c^Hexbio© granule with 3 ×10^10^ CFU/d	OMRON	72.2 ± 3.5 72.9 ± 2.6	Sedentary young males	2
	P, NR	9/12	100	21.4	12 weeks	^c^Hexbio© granule with 3 ×10^10^ CFU/d	OMRON	72.4 ± 3.1 71.7 ± 2.5	Circuit training for young males	2
Lefevre et al. (20) (France)	P, DB	50/50	21	63.2	16 weeks	2 ×10^9^ spores of *B. subtilis* CFU/d	No report	74.6 ± 12.2 69.6 ± 10.2	Healthy free-living elderly subjects	4
Möller et al. (21) (Pennsylvania)	P, DB	57/48	34	20.2	2 weeks	^d^VSL#3 with 112.5 billion CFUs a day	Critikon Dinamap Oscillometric Stationary Monitor	79.1 ± 11.5 78.9 ± 13.0	Young adults	3
Yang et al. (22) (China)	P, B	10/10	50	58.1	2 weeks	*Clostridium butyricum*, 420 mg/capsule, twice daily	No report	89.0 ± 8.5 90.0 ± 9.0	Laryngeal cancer patients	3
Ivey et al. (23) (Auatralia)	P, DB	40/37	57	68.0	6 weeks	Probiotic yogurt with *Lactis* Bb12 dose of 3.0 ×10^9^ CFU/d	Automated home blood pressure monitor	71.0 ± 9 71.0 ± 9	Overweight	5
	P, DB	39/40	58	65.0	6 weeks	Control milk with *Lactis* Bb12 dose of 3.0 ×10^9^ CFU/d	Automated home blood pressure monitor	70.0 ± 14.0 72.0 ± 12.0	Overweight	5
Jones et al. (24) (Canada)	P, DB	59/61	36	50.4	6 weeks	*Microencapsulated L. reuteri* NCIMB 30242 in a yogurt formulation with 5 ×10^10^ CFU, twice daily	No report	75.9 ± 4.8 75.4 ± 6.2	Mild hypercholester-olemic adults	4
Aihara et al. (25) (Japan)	P, DB	20/20	65	51.4	4 weeks	Powdered fermented milk with *L. helveticus* CM4, 12 g/day	UDEX-SUPER automatic sphygm-omanometer	71.9 ± 6.7 71.2 ± 8.0	High-normal blood pressure	4
	P, DB	20/20	80	51.7	4 weeks	Powdered fermented milk with *L. helveticus* CM4, 12 g/day	UDEX-SUPER automatic sphygm-omanometer	73.4 ± 6.3 73.4 ± 7.3	Mild hypertension	4

*P, parallel; B, blind; DB, double blind; TB, triple blind; NR, no reporting blind; CFU, colony forming unit*.

* *For parallel design, sample size and heart rate are intervention group/control group*.

a *Probiatop^®^ contains Lactobacillus para casei LPC-37, Lactobacillus rhamnosus HN001, Lactobacillus acidophilus NCFM, and Bifidobacterium lactis HN019 at a dosage of 10^9^ CFU of each strain*.

b *Hyperbiotics PRO-15 ADVANCED STRENGHT contains 15 different probiotics patented stains (Lactobacillus plantarum, Lactobacillus acidophilus, Bifidobacterium infantis, Lactobacillus fermentum, Lactobacillus reuteri, Lactobacillus casei, Bifidobacterium longum, Lactobacillus rhamnosus, Bifidobacterium lactis, Lactobacillus salivarius, Lactobacillus paracasei, Bifidobacterium bificum, Lactobacillus gasseari, Bifidobacterium breve, and Streptococcus thermophilus)*.

c *Hexbio granule contains six different microorganism strains (L. acidophilus BCMC^®^ 12130, L. casei BCMC^®^ 12313, L. lactis BCMC^®^ 12451, B. bifidum BCMC^®^ 02290, B. infantis BCMC^®^ 02129, and B. longum BCMC^®^ 02120)*.

d *VSL#3, freeze-dried probiotic bacteria, contains Bifidobacterium breve, B. longum, B. infantis, Lactobacillus acidophilus, L. plantarum, L. paracasei, L. bulgaricus, and Streptococcus thermophilus*.

With regard to participants, ten trials enrolled men and women, whereas four included only men and two only women. The mean age of participants ranged from 19.0 to 68.0 years old. The participants of nine trials were healthy, in other trials suffered from overweight (*n* = 2), hypertension (*n* = 2), hypercholesterolemia (*n* = 1), iron-deficiency (*n* = 1), and laryngeal cancer (*n* = 1). The baseline HR of participants in almost all trials was within the normal range from 68.8 to 90.0 bpm. Most of the studies reported no significant side effects of probiotics supplementation.

### Effect of Probiotics on HR

**Figure 2:**
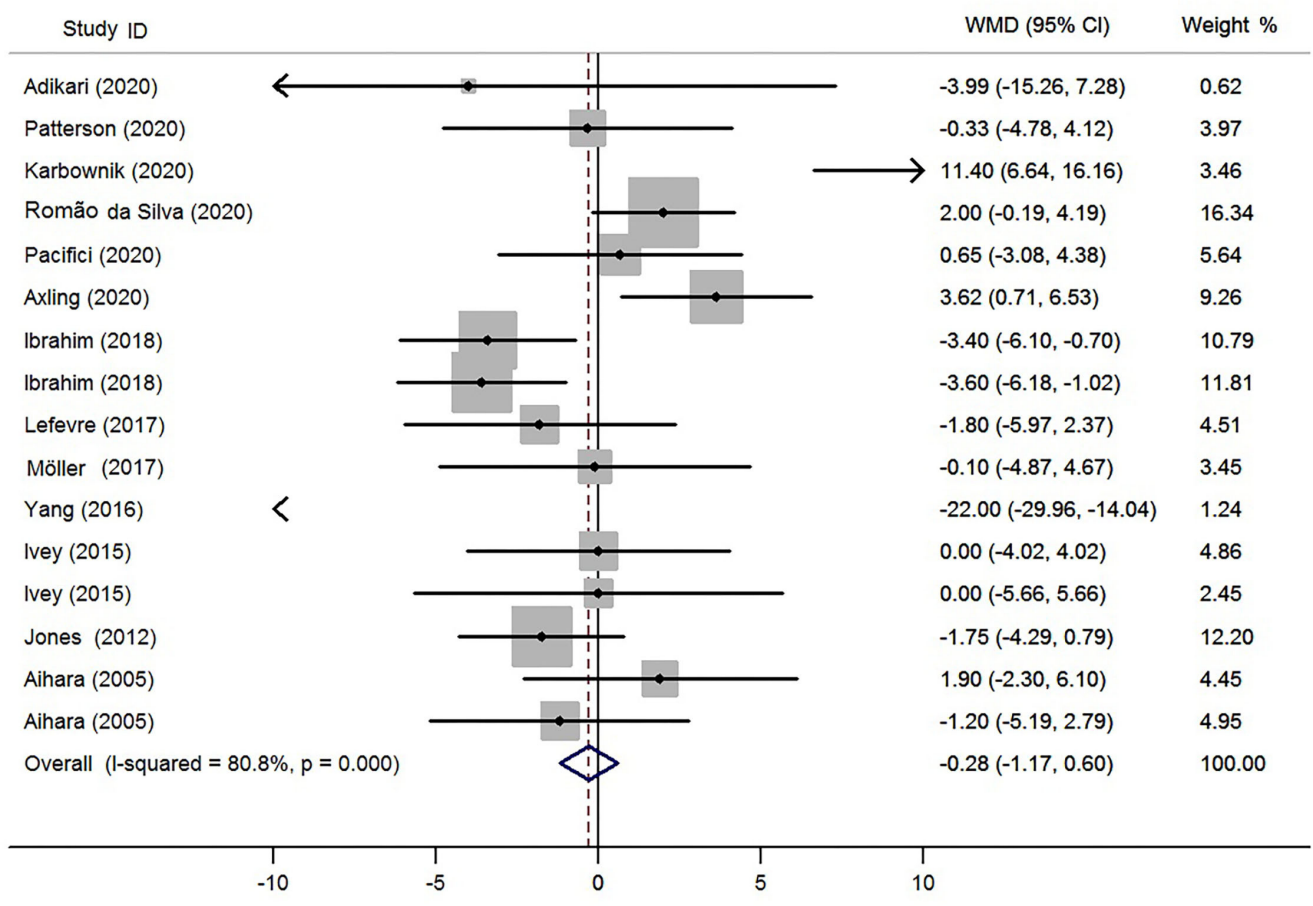
Meta-analysis of the effects of probiotics supplementation on heart rate (bpm). WMD, weighted mean difference.

The net changes in HR between the probiotics supplementation and control groups ranged from −22.0 to 11.4 bpm. Considering a great heterogeneity (*P* <0.01, *I*^2^ = 82.7%), the random-effect model was applied in the present meta-analysis. The pooled effect of probiotics supplementation on HR was −0.28 bpm (95% CI: −1.17 to 0.60; Figure 2), compared with the control group. This result indicated that probiotics supplementation had no effect on HR.

### Subgroup and Sensitivity Analysis

The results of subgroup analysis were shown in Table 2. Overall, probiotics supplementation produced a significant HR reduction among these trials at male participants (−2.70 bpm; 95% CI, −4.35 to −1.05), the mean age ≥ 45 years old (−1.57 bpm; 95% CI, −3.07 to −0.06), a probiotics dose ≥1 ×10^10^ CFU/day (−1.17 bpm; 95% CI, −2.34 to −0.00), and with a baseline HR ≥ 75 bpm (−2.94 bpm; 95% CI, −5.06 to −0.82) and a multiple-strain intervention (−1.43 bpm; 95% CI, −2.69 to −0.17). Inversely, probiotics produced a slight HR elevation at female participants (2.59 bpm; 95% CI, 0.83–4.34) and a probiotics dose <1 ×10^10^ CFU/day (2.03 bpm; 95% CI, 0.45–3.60). A sensitivity analysis was performed by omitting one trial each in turn to yield a narrow range from −0.73 (95% CI, −1.70 to 0.24) to −0.01 (95% CI, −0.90 to 0.88). The result suggested that no particular study significantly affected the overall findings for HR.

**Table 2:** Subgroup analyses according to trial and participant characteristics.

**Subgroup**	**No**	**Net bpm change**	***P*** **-heterogeneity**	***I*** **^2^ (%)**
		**(95% CI)**		
**Sex**				
Male	4	−2.70 (−4.35, −1.05)	0.276	22.4
Female	2	2.59 (0.83, 4.34)	0.384	0
Male and female	10	−0.36 (−1.67, 0.95)	0.000	83.6
**Mean age, year**				
<45	9	0.40 (−0.70, 1.50)	0.000	82.1
≥45	7	−1.57 (−3.07, −0.06)	0.000	79.2
**Study duration**				
<6 weeks	6	0.61 (−1.31, 2.52)	0.000	90.4
≥6 weeks	10	−0.53 (−1.53, 0.47)	0.003	63.7
**Health status**				
Generally healthy	11	−0.71 (−1.90, 0.47)	0.000	72.5
Disease status	5	0.26 (−1.07, 1.60)	0.000	90.1
**Baseline HR, bpm**				
<75 bpm	12	0.28 (−0.69, 1.26)	0.000	76.3
≥75 bpm	4	−2.94 (−5.06, −0.82)	0.000	87.6
**Form of probiotics**				
Capsule or sachet	10	0.10 (−1.15, 0.96)	0.000	88.0
Milk or yogurt or juice	6	−0.72 (−2.35, 0.91)	0.748	0
**Probiotics dose**				
<10^10^ CFU	5	2.03 (0.45, 3.60)	0.001	79.6
≥10^10^ CFU	8	−1.17 (−2.34, −0.00)	0.012	61.3
**Number of strains of probiotics**				
Single-strain	10	0.83 (−0.41, 2.08)	0.000	70.4
Multiple-strain	6	−1.43 (−2.69, −0.17)	0.000	87.9

### Meta-Regression Analysis

In order to explain the sources of heterogeneity, a meta-regression analysis was performed. To minimize the likelihood of false-positive results, we carefully selected a small number of covariates, including mean age, daily probiotics dose, and baseline HR. The present meta-analysis suggested that baseline HR was significantly associated with the effect estimate (*P* = 0.002) and accounted for 60.42% of the total between-study variation. By contrast, mean age and daily probiotics were not associated with a net change in HR (*P* = 0.486, 0.819). Therefore, baseline HR was considered as a major source of heterogeneity among trials.

### Publication Bias

Begg's test suggested no evidence of publication bias for the outcomes (*P* = 0.964). Egger's test also did not indicate evidence of publication bias (*P* = 0.586).

## Discussion

Probiotics are most frequently consumed in the forms of dietary supplements or beverages (31). The present meta-analysis comprehensively reviewed 13 RCTs with 16 intervention trial arms that evaluated the effect of probiotics administration as supplementation on HR in adults with or without diseases. To the best of our knowledge, this is the first meta-analysis exploring the relationship between probiotics supplementation and HR. The pooled effects based on the meta-analysis suggested that probiotics supplementation were insufficient to change HR. However, a higher probiotics dosage with more than 1 ×10^10^ CFU/day decreased HR by 1.17 bpm; a multiple-strain probiotics combination decreased HR by 1.43 bpm. We speculated that the negative chronotropic effect of probiotics on HR is dose-dependent and is enhanced when more strains are combined. In addition, the HR reduction by probiotics supplementation is profound in males or participants with the mean age ≥ 45 years old or baseline HR ≥ 75 bpm.

The present meta-analysis followed the PRISMA guidelines and had a relatively high Jadad score. However, it was limited primarily by considerable heterogeneity among studies, which complicated the interpretation of our findings. This phenomenon is perceivable because of the variation of the study characteristics. In trials included in the present meta-analysis, investigated strains were different; some administered single strain, others administered multiple strains. It is natural that each species and its metabolites interact with the host differently. Also, the participants included healthy adults, as well as patients with hypertension, hypercholesterolemia, iron deficiency, or laryngeal cancer. Health status may also differently affect the pharmacodynamics of probiotics on HR. In addition, genetic background or gene-diet interaction may partly explain the source of heterogeneity among studies. Due to the limited number of trials and the sample size, this meta-analysis did not further explore the effect based on these characteristics. The observed heterogeneity might be attributed to the two trials because the heterogeneity disappeared after the two trials were excluded in the sensitivity analysis. In the trial by Karbownik et al., the participants went through a stress test through a final academic exam, and HR was increased by probiotics supplementation (15). This increase may be partly attributable to the influence of a stress test before supplementation, because the stress may increase HR as an indirect marker of mental and physical stress (32). In the trial by Yang et al., the participants were a special population with laryngeal cancer and their baseline HR is the highest among all trials. In this trial, HR is surprisingly decreased by 22 bpm (22).

The results of subgroup analysis suggested the different influences of probiotics dose and strain on HR. HR reduction was more pronounced when probiotics supplementation with more than 1 ×10^10^ CFU/day or multiple probiotics strains, suggesting that probiotics dose and strain were two important factors influencing HR. In the definition of probiotics, it is required the administration of an adequate amount in order to obtain a health benefit. A systematic review also reported that probiotics with a larger dose daily exhibited a significant reducing effect on blood pressure and other diseases (33, 34). However, meta-regression analysis showed that probiotics dose was not a major source of heterogeneity among trials, so further trials with different doses are required to confirm these findings considering the small sample of trials in each subgroup. Furthermore, trials using multiple probiotics strains showed a statistically significant reduction in HR, while trials with a single strain had no effect on HR. The possible mechanism is attributed to the synergistic or additive effect of several probiotics; therefore, further studies are warranted to validate this finding.

Subgroup analysis results also indicated a significant reduction of HR in males or participants with the mean age ≥ 45 years old or baseline HR ≥ 75 bpm. Based on the meta-regression analysis, the baseline HR of subjects was a primary source of heterogeneity among trials, while the mean age was not. Although the baseline HR from the included trials was within the normal reference value, HR has been proved as an important marker of outcome in cardiovascular disease (35). From a Systolic Hypertension in Europe study, resting HR > 79 bpm was a significant predictor of all-cause and cardiovascular mortality in elderly patients with systolic hypertension (36). In our meta-analysis, HR with 75 bpm was acted as a cut-off point for subgroup analysis. A decrease in HR is helpful to reduce the risk of cardiovascular mortality. Thus, the present finding is useful, because probiotics supplementation might be effective in subjects with a high HR level.

Sub-diaphragmatic sensory nerves project centrally via the vagal afferents and dorsal spinal roots to reflexively regulate sympathetic or parasympathetic efferent nerve activities or tune their balance (37, 38). The favorable alteration of the gut microbial ecosystem is the most characterized biological function of probiotics (39, 40). Other than impacting the local environment, it regulates the excitability of the afferent nerves. The gastrointestinal afferents are an indispensable component of the microbiota-gut-brain axis in perceiving the information from the gut microbial ecosystem and then conveying it to the central nervous system (41, 42), which is another controlling mechanism on HR besides cardiac automaticity. From a dietary and nutrition perspective, the coupling between the gut-brain axis and the cardiac system is a new and interesting research field. Indeed, animal studies demonstrated that the change of gut microbial ecosystem either by prebiotics to proliferate probiotics population or by direct probiotics administration could prevent cardiovascular dysfunction by improving gut microbiota diversity and maintaining vascular integrity (43–45). In order to promote cardiovascular health and disease prevention to a wider population, further studies may identify and verify unique probiotics that may have better efficacy, safety, and feasibility profiles, and elucidate their mechanism of action in reducing HR.

In this meta-analysis, there are a few noteworthy limitations. First, relatively high heterogeneity was observed in all included trials. Therefore, the findings from this meta-analysis should be interpreted with caution. Considering the amount of the trials reporting no effect on HR, the observed heterogeneity across trials should be caused by the difference in statistical significance between trials rather than the difference in direction of the effect size. Second, the health status of participants varied widely across trials, and this might lead to overestimation or underestimation of the true intervention effect. However, the predefined subgroup and sensitivity analyses showed that the health status of participants did not affect the overall effect size. Third, the validity of our meta-analysis is dependent on the quality of the individual studies, but well-designed studies were not many. Specifically, allocation concealment, quality of randomization, and details of withdrawals were not always reported. Fourth, subgroup analysis showed that a higher probiotics dose can decrease HR. While this makes common sense, without having a dose-response curve established, it cannot be generalized now. Finally, the probiotics strain types investigated were diverse, which, on one hand, allows for cross-analysis of the relationship between more strains and the heart rate responses, on the other hand, disallowed for an accurate assessment of the efficacy of an individual strain.

## Conclusion

In conclusion, the evidence collected from published literature as of October 2021 was insufficient to prove the hypothesis that there is a class effect of probiotics supplementation on HR reduction in the overall analysis. Interestingly, however, probiotics reduced HR significantly in males, and in those who were relatively older (≥45 years) or with higher baseline HR (≥75 bpm), received a higher dose (≥10^10^ CFU/day), or multi-strain of probiotics in subgroup analysis. Meta-regression analysis demonstrated that baseline HR was a potential effect modifier and a major contributor to the overall between-study variation, therefore, the present results should be interpreted with caution because of the existence of heterogeneity. Taken together, it is justified to pursue future investigations on a larger scale, with an adequate dosage of promising strain(s), and with a targeted population(s) for the purpose of HR reduction, which will determine if some probiotics constitute an effective, safe, and applicable dietary or nutrition approach in the strategic framework of lifestyle modification for cardiovascular risk control and disease prevention.

## Data Availability Statement

The original contributions presented in the study are included in the article/Supplementary Material, further inquiries can be directed to the corresponding author/s.

## Author Contributions

SH and WZ: study design and analysis and interpretation of data. SH, YL, and HG: literature search. SH, YL, and RS: data collection, extraction, and quality assessment. SH: writing-original draft. WZ: writing-review and editing. All the authors reviewed and approved the final manuscript.

## Funding

This study was supported by Scientific Research Foundation for Scholars of HZNU (No.2022QDL004).

## Conflict of Interest

The authors declare that the research was conducted in the absence of any commercial or financial relationships that could be construed as a potential conflict of interest.

## Publisher's Note

All claims expressed in this article are solely those of the authors and do not necessarily represent those of their affiliated organizations, or those of the publisher, the editors and the reviewers. Any product that may be evaluated in this article, or claim that may be made by its manufacturer, is not guaranteed or endorsed by the publisher.
